# Association between the Type of Workplace and Lung Function in Copper Miners

**DOI:** 10.1155/2016/5928572

**Published:** 2016-05-05

**Authors:** Anna Skoczyńska, Leszek Gruszczyński, Anna Wojakowska, Marek Ścieszka, Barbara Turczyn, Edward Schmidt

**Affiliations:** ^1^Department of Internal and Occupational Diseases and Hypertension, Wrocław Medical University, Borowska 213, 50-556 Wrocław, Poland; ^2^The Copper Health Center, M. Skłodowskiej-Curie 66, 59-301 Lubin, Poland

## Abstract

The aim of the analysis was to retrospectively assess changes in lung function in copper miners depending on the type of workplace. In the groups of 225 operators, 188 welders, and 475 representatives of other jobs, spirometry was performed at the start of employment and subsequently after 10, 20, and 25 years of work. Spirometry Longitudinal Data Analysis software was used to estimate changes in group means for FEV_1_ and FVC. Multiple linear regression analysis was used to assess an association between workplace and lung function. Lung function assessed on the basis of calculation of longitudinal FEV1 (FVC) decline was similar in all studied groups. However, multiple linear regression model used in cross-sectional analysis revealed an association between workplace and lung function. In the group of welders, FEF75 was lower in comparison to operators and other miners as early as after 10 years of work. Simultaneously, in smoking welders, the FEV_1_/FVC ratio was lower than in nonsmokers (*p* < 0,05). The interactions between type of workplace and smoking (*p* < 0,05) in their effect on FVC, FEV1, PEF, and FEF50 were shown. Among underground working copper miners, the group of smoking welders is especially threatened by impairment of lung ventilatory function.

## 1. Introduction

Copper miners working underground are exposed to mine gases containing, among other things, nitric oxides. Endogenous nitric oxide (NO) plays key roles in lung biology, being involved in pulmonary neurotransmission, as well as host defense, airway and vascular smooth muscle relaxation, mucociliary clearance, airway mucus secretion, inflammation, and cytotoxicity [1, 2]. Simultaneously, endogenous NO has been implicated in the pathophysiology of lung diseases [3]. The production of NO under oxidative stress conditions generates strong oxidizing agents (reactive nitric species) that may influence the development and the course of chronic inflammatory airway diseases such as asthma, cystic fibrosis, bronchopulmonary dysplasia, lymphangioleiomyomatosis, and pulmonary hypertension [4–7].

On the other hand, the respiratory system is known to be critical for toxicity of exogenous nitric oxides. Nitric dioxide (NO_2_) and nitric oxide, dissolving in water contained in the bronchial mucus, form nitric acid and nitrous acid. These acids may produce acute pulmonary injury due to inhalation of vapors and gases originating from nitric acid solutions. Acute poisoning may occur as a result of short-term exposure to nitric oxides at high concentrations (94–7500 mg/m^3^) in the course of mining disasters, breakdowns, and accidents. Nitric oxides in very high concentrations can cause immediate death, toxic pulmonary edema occurring within 48 hours, pneumonia [8], or acute respiratory distress syndrome [9]. By combining with the alkaline substances contained in the mucous secretion, a part of nitric and nitrous acids is converted into nitrite, which may cause methemoglobinemia [10]. Thus, the consequence of acute or chronic exposition to nitric oxides is respiratory failure from hypoxemia and, next, hypercapnia. The result of chronic, repeated exposure to nitric oxides can be fibrous chronic bronchitis, diffuse interstitial lung fibrosis with emphysema, and bronchial hyperreactivity. It was documented that chronic, occupational exposure to nitric oxides is associated with increased susceptibility to respiratory infections [11] and with changes in spirometric indices [12, 13]. The toxic effect of nitric oxides is investigated in view of the recent debate on European limits, recommended by the Scientific Committee on Occupational Exposure Limit Values (SCOEL) [14, 15].

Few studies have shown that miners working under the ground may be chronically exposed to various levels of nitric oxides. The sources of NO_2_ and NO are mine gases, diesel engine emissions, welding technology, the use of explosives for blasting, and also smoking [16, 17]. The aim of this analysis was to assess changes in lung function in copper miners potentially exposed to nitric oxides over 25 years of work, based on the results of repeatedly performed spirometry tests. Another goal was to examine whether lung dysfunction in underground miners is dependent on the type of workplace.

## 2. Material and Methods

### 2.1. Workers

Results of spirometric tests, which have been conducted in 888 miners during their 25 years of employment at the same copper mine, between 1980 and 2005, in Poland, in Lower Silesia, were analyzed. Miners were working underground as operators (225 men), welders (188 men), and other workers (475 men): mechanics, electricians, transport workers, and hewers (miners employed in the direct obtaining of excavated material). The samples of mine air were taken every month to determine time weighted average (TWA) and a short-term exposure limit (STEL) for nitric oxides. The determined TWA concentrations ranged from subdetectable (most often) to 2,02 mg/m^3^ (rarely in 2001–2009), that is, below Maximum Allowable Concentration (MAC). The short-term exposure limit for nitric dioxide (1,5 mg/m^3^) was sometimes exceeded and in some measurements in 2007 reached as much as 3,96 mg/m^3^.

Anthropometric data (body weight and height) were collected in local health centre, using calibrated equipment and standardized methodology. Body mass index (BMI) was estimated as the ratio of weight to height squared (kg/m^2^).

Spirometry was performed on workers four times: at the start of employment (between 1980 and 1983) and then 10 years after (between 1989 and 1991), 20 years after (between 1998 and 2000), and 25 years after the beginning of work (between 2003 and 2005). The procedures were in accordance with ethical standards and the Helsinki Declaration of 1975, as revised in 1983.

At the time of the first spirometry, among the miners, 435 men were nonsmokers and 354 were smokers (average rate: 20 cigarettes × 8,1 ± 5,6 years of smoking), and in 99 cases there was no information on smoking. A respiratory disease was diagnosed in 15 workers: pneumoconiosis (in eight miners) or allergic respiratory disease (in seven workers). There were no data on respiratory diseases in 108 men. In some groups of workers, results of blood count were analyzed. The number of miners, including smokers and men with respiratory disease, has changed over the period of employment, as shown in Table 1. The largest decrease in the number of obtained spirometry results concerned the study that was performed in 1998–2000 and was due to the loss of part of spirometry tests' results during transfer of the archive. The biggest number of missing results (50%) concerned the group of “other” miners. However, in groups of operators and welders, losses were significantly lower (2.6% and 23%, resp.), so in the analysis (except for the analysis using the SPIROLA program) also the data from 1998–2000 was taken into account.

### 2.2. Equipment and Interpretation

Spirometry was carried out at first according to American Thoracic Society criteria (as described in [18]) and then, since 1993, according to ERS guidelines (published in [19]). Tests were performed on Spirolab spirometer (Roma, Italy). Patients were assessed for contraindications to spirometry; data on the height, body mass, and other required parameters (age, gender, and ethnicity) were collected. The procedure was explained to the patient, a nose clip was applied, and a minimum of three acceptable VC manoeuvres were obtained. Repeatability criteria were met when there was no more than 100 mL ideally (and certainly no more than 150 mL in the occasional highly variable patient) between each blow. Patient was verbally encouraged to continue to exhale as long as possible and, next, VC manoeuvres (no more than four) and FVC manoeuvres (a minimum of three acceptable, maximum eight) were obtained. The best value was recorded for forced expiratory volume in the first second (FEV_1_), forced vital capacity (FVC), peak expiratory flow (PEF), and four parameters of forced expiratory flow (FEF): FEF_25_, FEF_50_, FEF_75_, and FEF_25–75_. The mean FEV_1_/FVC ratio (forced expiratory volume in the first second, expressed as a percentage of the forced vital capacity) was calculated. The technical accuracy of performed spirometry tests and the precision of collected data were monitored by technicians trained in ATS standards. Inspection of flow-volume curves served to control complete exhalation, indicated by gradual flow drop to zero. Interpretation of the results was based on guidelines published in 2005 by the experts of the American Thoracic Society (ATS) and European Respiratory Society (ERS) concerning the correctness of the implementation and evaluation of spirometry [20, 21]. In case of FEV_1_, FVC, and FEV_1_/FVC, a lower limit for normal value (LLN) which corresponds to the fifth percentile in healthy nonsmokers and/or a lower limit for normal decline were estimated [22]. NIOSH (National Institute for Occupational Safety and Health) Spirometry Longitudinal Data Analysis (SPIROLA v3.0.2) software was used, which monitors group means for FEV_1_ and FVC in relation to mean predicted values based on group demographics (age, height, gender, and ethnicity/race). For the first 8 years of follow-up, SPIROLA uses the limit of longitudinal decline (LLD) which takes into account expected within-person variation in FEV_1_ or FVC and the duration of follow-up. After 8 years of follow-up, the age at which the individual is projected to develop lung function impairment is considered in the evaluation. Using SPIROLA program, we also evaluated whether FEV_1_ and FVC slopes for studied cohort exceeded 40 mL per year (referential date for decline using within-person standard deviation of 4%) and, on the basis of risk list function, determined how many subjects met criteria for abnormality.

### 2.3. Statistical Analysis

The impact of the type of workplace on lung function in copper miners was investigated as the main objective of this analysis. The independent variable was defined as operators, welders, or “others” and was individually analyzed in relation to each lung function outcome in the multiple linear regression model. This analysis was performed retrospectively fourfold, including results of spirometry obtained at the start of employment and after 10, 20, and 25 years of work. The following variables were included as potential confounders in the analysis of lung function: age, height, weight, BMI, presence of respiratory disease, and smoking status.

Spirometric indices were presented as mean ± SD, median and IQR, and percentile: 5% and 95%. Depending on the type of variable distribution, parametric or nonparametric methods of analysis were used. In the case of normal distribution, *t*-tests including paired test for dependent samples were applied, and a statistical significance between means was calculated using ANOVA test. In case of qualitative variables, nonparametric tests were used. Two-way analysis of variance (using workplace and smoking as independent factors) was also applied with one- or multidimensional significance tests. Correlations between variables were checked using Spearman coefficient. *p* value less than 0,05 was accepted as statistically significant. Analyses were carried out using STATISTICA version 12.0.

## 3. Results

The mean height of operators, welders, and other miners was similar. At the beginning of employment, the median BMI values were greater than 25.0 kg/m^2^ in all groups. At this time, BMI was greater than 30.0 kg/m^2^ in 113 miners and greater than 35.0 kg/m^2^ in 13 men. BMI in welders was higher than in remaining miners. Changes in body mass index during 25 years of work were similar in all groups of workers, whereas the age differences were not similar (Table 1). Among studied groups of miners, operators were younger, on average by 3–5 years, in comparison to welders and representatives of other jobs. The inverse linear relationship between age and FEV_1_/FVC ratio was shown (*r* = −0,1361; *p* = 0,0000). In the group of miners studied after 20 years of work, the inverse relationship between age and FVC (*r* = −0,1971; *p* < 0,0000) and between age and FEV_1_ (*r* = −0,1734; *p* < 0,0000) was observed.

### 3.1. Changes in Spirometric Parameters over 25 Years of Work

In spirometry tests performed at the start of the employment and after 10, 20, and 25 years, in each group (operators, welders, and others), the mean FEV_1_/FVC ratio was greater than 70% (Table 2). However, in the first spirometry, in 21 (10%) operators, 12 (6%) welders, and 26 (5%) other miners (in 59 (6,6%) workers in total), FEV_1_/FVC was lower than LLN. In this subgroup of men with reduced FEV_1_/FVC ratio, in ten miners (6 operators, 2 welders, and 2 others), also FEV_1_ was less than LLN. With the expiration of the employment period, the number of miners with obstructive disorders decreased; after 10 years, it was 31 men, after 20 years 26 men, and after 25 years 30 men. The number of miners with both FEV_1_/FVC and FEV_1_ lower than LLN was 3, 4, and 7 after 10, 20, and 25 years of work, respectively.

**Figure 1:**
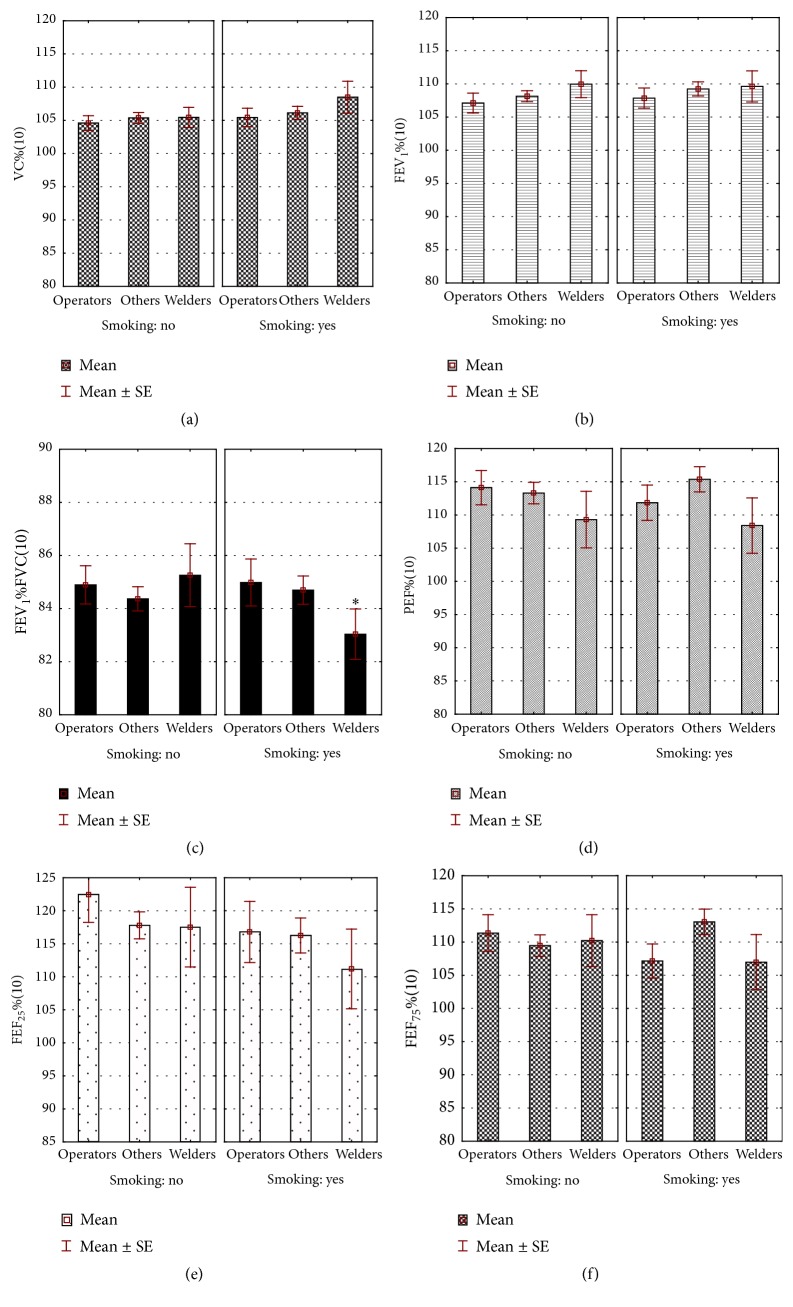
Spirometric parameters in miners after 10 years of work depending on the workplace and smoking. ^*∗*^Statistically significant differences between nonsmokers and smokers; *p* < 0,05.

**Figure 2:**
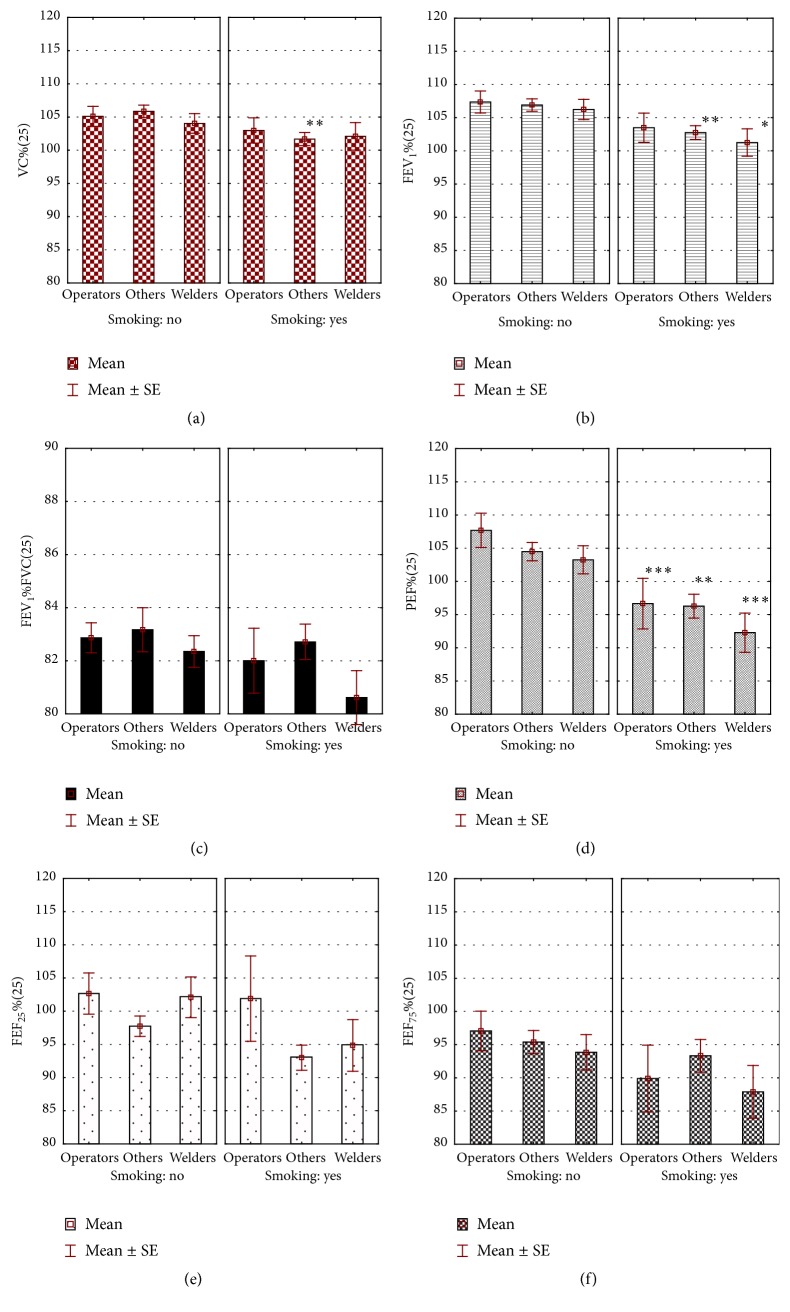
Spirometric parameters in miners after 25 years of work depending on the workplace and smoking. ^*∗*, *∗∗*, *∗∗∗*^Statistically significant differences between nonsmokers and smokers; ^*∗*^ *p* < 0,05; ^*∗∗*^ *p* < 0,01; ^*∗∗∗*^ *p* <0,001.

*P*-paired test for dependent variables performed in the whole group showed a gradual, significant decrease in FEV_1_/FVC ratio over a 25-year period of occupation (Table 2). The mean FEV_1_ (4,096 ± 0,72 L initially) decreased in subsequent years to 3,825 ± 0,71 L. Using SPIROLA software, it was shown that the mean rate of FEV_1_ absolute decline from first to last measurement was 10.0 mL/year (relative decline was 0.3%/year), and mean slope for FEV_1_ (LLD) amounted to −12.4 mL/year (relative slope: −0.3%/year), with root mean square error (RMSE) of 117 mL. Simultaneously, in the course of performed work, a decrease in % PEF, % FEF_25_, and % FEF_75_ was shown (Figure 1 versus Figure 2).

### 3.2. The Analysis of Spirometric Indices Depending on the Type of Underground Work

**Figure 3:**
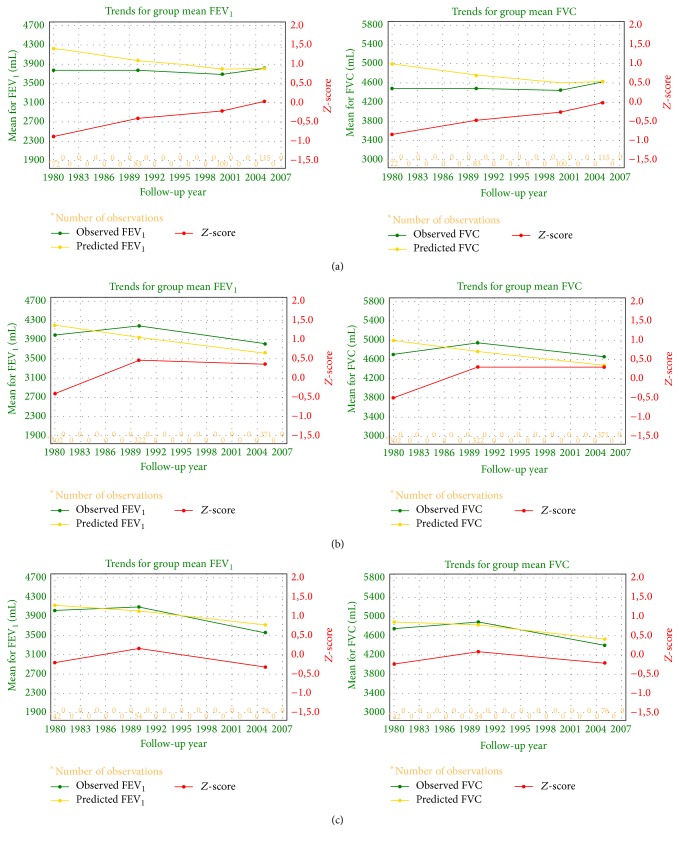
Trends in FEV_1_ and FVC over time in operators (a), others (b), and welders (c). On the basis of SPIROLA v3.0.2 software. The mean FEV_1_ chart shows group means for observed, predicted, and *Z*-score (standard deviation units from the predicted quantity) values. The same applies for FVC chart. The predicted values are derived from prediction equations that take into account age, height, sex, and race/ethnic background and are based on nationally representative healthy never-smokers. Irregular deviations of observed mean values from predicted values may be due to changes in measurement procedures or due to effects of occupational exposure or interventions.

In group of copper miners employed as operators, during 25 years of follow-up, the mean group slope was −1,0 mL/year, with within-person variation for the group (root mean square error, RMSE) of 329 mL, whereas mean slope for FVC was 10 mL/year, with RMSE of 127 mL. In the same period, in group of welders, mean group slope for FEV_1_ was −21 mL/year, with RMSE of 540 mL, and mean slope for FVC was −14 mL/year, with RMSE of 532 mL. In group of miners employed at other workplaces, mean group slope for FEV_1_ was −8,0 mL/year with RMSE of 535 mL, and for FVC it was 0,0 mL/year, with RMSE of 603 mL. In risk list of SPIROLA, excessive variation for FEV_1_ (>10,1%) and/or FVC (>7,7%) was present in 28% of operators, 17% of welders, and 24% of other miners. Trends in FEV_1_ and FVC over time in each group of miners were presented in Figure 3.

**Figure 4:**
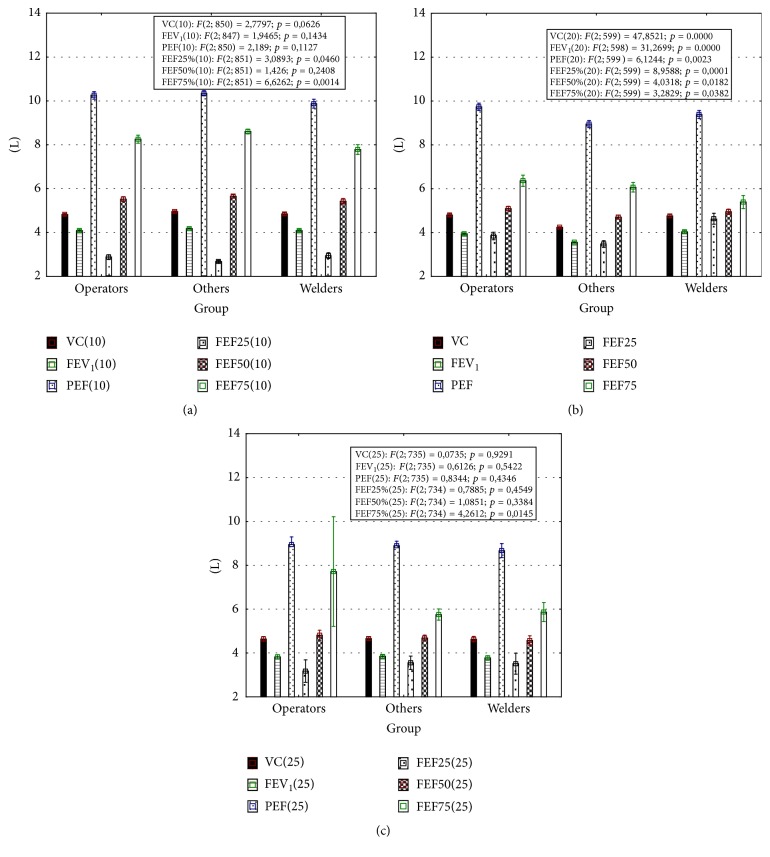
Absolute spirometric indices after 10 (a), 20 (b), and 25 (c) years of work. Results are presented as mean ± standard error. In frames results of ANOVA *F*-test were presented. This test was used to assess whether any of the workplaces is on average superior, or inferior, to the others versus the null hypothesis that all three workplaces yield the same mean response in lung function.

Significant differences in spirometric parameters between welders and other job's representatives appeared after 10 years of employment. In welders, the values of PEF (9,8 ± 2,7) and FEF_75_ (7,8 ± 2,9) were lower in comparison to group of other workers (10,3 ± 2,5; *p* < 0,05 and 8,6 ± 2,4; *p* < 0,001, resp.) (Figure 4(a)). Simultaneously, multiple linear regression analysis did not show any significant association between workplace and lung function (Table 3).

After 20 years of work, significant differences between studied groups of workers included all spirometric indices (Figure 4(b)). Except for FEF_75_, these parameters were lower in the group called “others,” in comparison to the group of operators or welders. Multiple linear regression analysis adjusted for age, height, weight, BMI, smoking, and presence of respiratory disease showed an association between workplace and lung function. The significant beta-coefficients for FVC, FEV_1_, FEF_25–75_, and FEF_50_ have been demonstrated (Table 3).

After 25 years of work, welders displayed significantly (*p* < 0,05) lower % PEF (99,0 ± 22,7), in comparison to operators (104,1 ± 26,1) and others (101,7 ± 23,0). The mean FEF_25_ values in welders (5,9 ± 2,8) and “others” (5,7 ± 2,7) were lower than in operators (7,7 ± 5,2), *p* < 0,05 and *p* < 0,01, respectively (Figure 4(c)). Association between type of workplace and lung function parameters (FVC and PEF) was documented (Table 3).

Among all confounding factors, height and absolute values of BMI had the highest positive beta-coefficients in a linear regression model (i.e., in the group of miners studied after 20 years of work, the coefficient of correlation between BMI and FVC reached the value of 0,950), whereas presence of respiratory disease had the lowest negative coefficients. The analysis of spirometric parameters depending on the coexisting respiratory disease showed that % FVC in the subgroup of 15 miners diagnosed with respiratory disease was lower (*p* < 0,05), in comparison to healthy workers (91,5 ± 8,7 versus 100,9 ± 13,7 in initial spirometry; 97,6 ± 10,3 versus 105,7 ± 13,8 after 10 years; and 98,0 ± 10,1 versus 105,9 ± 17,2 after 25 years of work).

### 3.3. The Analysis of Spirometric Indices Depending on Smoking

Among the confounding factors, the status of smoking had the greatest variability. Therefore, the analysis was extended by excluding smoking from the group of confounders and looking for existence of interaction between the workplace and smoking as independent factors.

The differences between smokers and nonsmokers were displayed at the start of employment. At that time, the mean period of smoking was 8,1 ± 5,6 years. The values of FEV_1_ and PEF, expressed as percentage of predicted normal values, were significantly (*p* < 0,05) lower in smokers than in nonsmokers (% FEV_1_: 102,9 ± 15,2 and 105,6 ± 14,9; % PEF: 106,4 ± 25,8 and 110,1 ± 26,9, resp.). After 10 years of work, lower FEV_1_/FVC in smoking, in comparison to nonsmoking welders, was shown (Figure 1(c)). After the next 15 years, in general, spirometric parameters were also lower in smokers than in nonsmokers, and they were the lowest in the group of smoking welders (Figure 2).

The interactions between workplace and smoking in their effect on FVC, FEV_1_, % PEF, % FEF_25_, FEF_50_, and % FEF_75_ appeared after 20 years of work. Five years later, such interactions concerned only % FVC; however, smoking independently influenced the FEV_1_/FVC ratio, % PEF, and % FEF_75_ (Table 4).

## 4. Discussion

Commonly accepted indications to spirometry tests are diagnostic, epidemiological, and related to monitoring and to certification [21]. All these indications were present in underground copper miners exposed to nitric oxides and other mine gases. In this retrospective analysis of spirometric parameters, the impact of type of workplace on lung function over 25 years of observation was estimated.

### 4.1. Analysis of Spirometric Parameters in View of Bronchial Obstruction

An obstructive ventilatory disorder is a disproportionate reduction of maximal air flow from the lung in relation to the maximal volume that can be displaced from the lung. In the first spirometric test performed in 888 workers, the most important indicator of bronchial obstruction, the FEV_1_/FVC ratio, was normal according to recommendation of expert panel [23, 24], that is, higher than 70% of the predominant value. In recent years, it has been assumed that FEV_1_/FVC index equal to 70% does not reflect the real lower limit of the norm and it can be used only as a screening criterion or eligibility criterion for further diagnosis [25–27]. In 25 years of follow-up, it could be expected that a greater proportion of the population would have the FEV_1_/FVC ratio below 70%, as the lower limit of normal (LLN) FEV_1_/FVC ratio would fall slightly with age. Therefore, it was better to refer obtained results to LLN, which includes age, than to fixed values. FEV_1_/FVC reduced below LLN was shown in the first spirometry in about 7% of subjects: in about 5% of these miners, the mean FEV_1_ was greater than LLN, and thus mild obstruction could be diagnosed, whereas, in 1% of miners, FEV_1_/FVC index and FEV_1_% were lower than LLN, leading to a diagnosis of moderate bronchial obstruction. Among these subjects, there were smokers (nearly 45%) and men treated due to respiratory diseases. In relation to these data, the occurrence of bronchial obstruction in a few percent of miners appears to be only a small percentage of the total number of studied workers. However, it should be an indication to intense preventive activity, especially concerning smokers and miners diagnosed with respiratory disease.

After 10 years of employment, the number of miners with a reduced FEV_1_/FVC index was about twice lower (3%) than in the first spirometry. After 20 years, bronchial obstruction could be diagnosed in 4,3% of miners and after 25 years in about 4%. A decreased number of men with low lung function during 25 years of observation, associated with small number of miners diagnosed with a respiratory disease, could be a consequence of the decreased percentage of smokers in the studied population. On the other hand, miners with a decreased FEV_1_/FVC index displayed gradual decrease in % FEF_25_ and % FEF_75_, which indicates the impairment not only of central, but also of peripheral bronchial function. It is documented that obstructive changes may be manifested as a reduction of air flow in the final phase of exhalation, which results in the maximum expiratory flow disturbances, especially at FEF_25–75_, FEF_50_, and FEF_75_. These changes are nonspecific, and the variance of these indicators in a healthy population is very large; hence, the reduction does not constitute a basis for diagnosis of pathology in case other results are correct [26]. Many somatic parameters, as gradual body mass increase, observed also in our study, can influence these spirometric changes [28]. Moreover, progressive age of workers impacted, alongside the length of employment period, lung function, as the negative correlation between age and FEV_1_/FVC ratio (*p* < 0,001) was shown in our study, similarly as in others [29].

Additionally, in comparison to the first spirometry, spirometric tests conducted after 25 years of the employment showed a significant decrease in the relative peak expiratory flow. The reduction of PEF is a characteristic form of bronchial obstruction in the course of chronic bronchitis or asthma [30], whereas in this study allergic disease was diagnosed only in seven workers. On the other hand, % PEF changes did not exceed 10% in relation to initial values. PEF is known as a good index of the bronchi patency but is subject to oscillation; that is, in healthy men, even a short-term (i.e., twenty-four hours) fluctuation of PEF reaches 15%.

### 4.2. Assessment of Spirometric Indices in View of Restriction

In the absence of the measurement of total lung capacity, it is acceptable to classify the degree of restriction on the basis of the FVC; however, it is not synonymous with a diagnosis of restriction. Correct FVC values do not exclude the possibility of functional disorders concerning mechanical properties or diffusion across the alveolar-capillary barrier [23, 24]. Our analysis showed a reduced % FVC below the 5th percentile of FVC only in 4.4% of workers. Reduction in FVC% was accompanied by a slight (statistically nonsignificant) decrease in FEV_1_/FVC ratio, so the occurrence of restrictive changes in this group could be suspected. However, taking into account the simultaneous reduction in FEV_1_ (to value less than LLN), the number of people with suspected restrictive changes decreased to 9 (1.0%). After 10 years of work, the percentage of such workers was 0.6%, after 20 years, 1.2%, and after 25 years, 2.9%. Regarding the entire pool of workers, the problem of restrictive changes seems to be important only individually.

### 4.3. The Impact of the Type of Workplace on the Lung Function

During 25 years of observation, the decrease in FEV_1_/FVC ratio and FEV_1_ was observed in all groups of copper miners. Absolute and relative decline in FEV_1_ from first to last measurement were similar in operators, welders, and other miners and were much less than 40 mL/year (referential data). Nonetheless, the mean group slope for decline of FEV_1_ in welders (−21 mL/year) was greater in comparison to operators (−1 mL/year) or other workers (−8 mL/year). The rate of decline < 30 mL/year, established over 5 or more years, is not associated with increased morbidity and mortality regardless of the level of lung function [31]. Also, the absolute and relative slopes for FEV_1_ were similar in all studied groups of miners and amounted to less than 15% in long-term observation. The American College of Occupational and Environmental Medicine [32] recommends a longitudinal limit based on annual acceptable decline of 15%, which is comparable to LLD, using within-person variation of 6%. Trends in FEV_1_ and FVC, showing prognosis for subsequent years, were also similar in groups of operators, welders, and other miners. Thus, respiratory function assessed on the basis of the mean slope and within-person variation for FEV_1_ (FVC) in studied groups was similar in miners working underground at various workplaces. Impaired lung function, assessed on the basis of excessive FEV_1_ (FVC) decline, occurred in such a small number of miners that it was difficult to state whether there was increased predisposition of miners employed at any particular workplace.

Simultaneously, using the multiple linear regression model in cross-sectional analysis, the association between workplace type and lung function, independent of the impact of age, height, weight, BMI, smoking, and coexisting respiratory disease, was shown. This association appeared after 20 years of underground work. However, differences in spirometric indices between welders and other miners were apparent as early as after 10 years of work. At that time, welders displayed a slightly lower % PEF and a significantly lower % FEF_75_ than operators or other employees. These results can announce obstructive disturbances rather than restrictive ones. Also, Fidan et al. observed a decrease in parameters of forced expiratory flow, including FEF_75_, and, simultaneously, a higher risk for chronic bronchitis in welders in comparison to a control group [33]. In case of welders working underground, apart from the impact of mine gases containing nitrogen oxides, an additional source of emissions of these gases, as well as harmful metal oxides particles, is welding fumes [34]. This complex exposure may explain the occurrence of early obstructive changes in welders. In our analysis, lung function disorders in welders were dependent upon the time of exposure; they first appeared after 10 years of employment and expanded in the following measurements to other indices, including significant changes in FEV_1_. Also, other authors indicated a reduction in FEV_1_ or PEF in welders exposed to welding gases for more than 9 years, whereas workers with exposure of less than 5 years did not show any significant changes [35]. Haluza et al. observed a decrease of pulmonary function during the period of occupational exposure to welding fumes [36]. Among British coal miners, the loss of FEV_1_ over 11 years was related to occupational exposure and to smoking [12].

After 20 and 25 years of work, association between workplace type and lung function was shown as dependence of absolute spirometric parameters on type of job in a linear regression model. These parameters included indicators of not only obstruction, but also restriction. In comparison to welders or operators, other workers displayed lower lung function. These results can be a consequence of long-term exposure to mine gases containing nitric oxides as well as other gases toxic for the respiratory system, that is, hydrogen sulfide. The group of “other” workers included hewers who are the most exposed workers to dust and gases among miners.

In general, during the 25 years of this follow-up observation, the exposure of miners to nitrogen oxides, especially nitrogen dioxide, was low. The time weighted average (TWA) concentrations for nitric oxides ranged from subdetectable (most often) up to 2,02 mg/m^3^ (rarely in 2001–2009), which is below respective Maximum Allowable Concentrations (MAC). The short-term exposure concentration (STEL) for nitric dioxide in some cases exceeded safe levels and in some measurements in 2007 reached 3,96 mg/m^3^. In 2002, MAC and STEL values were corrected according to the SCOEL recommendation (at present, the MAC amounts to 0,6 mg/m^3^ for NO_2_ and 3,0 mg/m^3^ for NO, whereas the STEL for NO_2_ is 1,5 mg/m^3^ and for NO is 7,5 mg/m^3^) [14].

In the analysis, the impact of confounding factors on lung function must not be omitted. In each group, the negative effect of age was typical [22, 29], while the effect of height and BMI in relation to spirometric parameters was similar to the one observed by other authors in normal-weight subjects [37–39]. In most miners, the measured BMI gradually increased over 25 years of observation; however, in most cases, it increased up to value lower than 30,0 kg/m^2^. It may explain the positive correlation observed between BMI and FVC. Also, Chen et al. observed that, in subjects with normal weight, BMI was positively associated with FVC and FEV_1_ [40].

Another reason for the moderate increase in FVC could be the decrease in number of smokers during 25 years of work. On the other hand, smoking remains the single most important cause of obstruction [41]. In our study, differences in obstructive indices between smokers and nonsmokers appeared as early as in the first spirometry. The mean period of smoking was then about eight years. In comparison to nonsmokers, smokers displayed a significant decrease in % FEV_1_ and % PEF, recognized as sensitive indicators of reduced air flow through constricted bronchi [42]. As might be predicted, over years of smoking, the differences between smokers and nonsmokers expanded to other spirometric indices, and, after 10 years of work, a significant lower FEV_1_/FVC index in smokers was shown. The mean age of smoking workers was then 38,5 ± 3,61 years. Throughout the 25 years of the study, the biggest decline of lung function was observed in smoking welders (Figures 1-2), as well as in other studies [43, 44].

According to the National Lung Health Education Program, primary care should perform spirometry tests on smoking patients aged 45 years or older in order to detect airways obstruction and aid smoking cessation efforts [45]. These recommendations were developed based on the research conducted over 20 years, which summarizes the results of spirometry, smoking status, and symptoms of respiratory disease in the US population. Air flow obstruction was defined on the basis of the abnormal value of FEV_1_/FEV_6_ and FEV_1_. Such changes in spirometry in smokers were revealed mainly in subjects aged over 45 years, and only 5 percent of smokers aged less than 45 years showed abnormal ventilation. Our analysis showed the age of smokers with worse lung function to be lower by a few years. This difference may result from specificity of miners work conditions, including underground work in exposure to mine gases and dust. Of much significance could be the existence of an interaction between workplace and smoking, confirmed by this analysis in workers working for 20 years or more. On the other hand, our results confirm the validity of European societies' recommendations to evaluate the effects of smoking in male smokers who are under 45 years of age and also the relevance of the National Program of Early COPD Detection [46], advising to evaluate the effects of smoking in smokers who are under 40 years old.

### 4.4. Advantages and Disadvantages

An advantage of this study is the diversification of the professional group of copper miners, which allowed an evaluation of the association between type of workplace and lung function in cross-sectional analysis. The groups of miners were numerous; moreover, miners were monitored over a long period of 25 years, which allowed the assessment of the relationship between workplace and lung function in this longitudinal observation.

One limitation of this analysis is the lack of a control or low to no exposure group for comparison. The disadvantage of the study may be a various number of miners in successive stages of observation. This has resulted in a reduced number of miners investigated in next stages of this evaluation, but especially between 1998 and 2000. It has been reported that results from longitudinal study of workers may be biased if there is a systematic exodus of certain types of workers between the surveys [12]. Other survey bias that can significantly influence the results, that is, a significant increase in lung function drops at a particular testing site or during a particular testing time period, has been described [47]. In this study, data for miners who attended the first survey were not separated into those collected from subjects who also attended the later survey (stayers) and those taken from subjects who did not (leavers), as in many individual cases results of only one test (usually the test performed after 20 years of start of employment) were unavailable. However, a variable number of available spirometry results could affect the accuracy of analysis. Assessment of lung function decline is affected also by factors such as spirometry tests' technical quality, test variability, testing frequency, duration of follow-up, and definition of excessive decline [26]. The precision of longitudinal measurements has been often determined by the magnitude of the within-person variation. In this study, using SPIROLA program, the authors monitored retrospectively the magnitude of the within-person relative standard deviation *s*_*r*_ for FEV_1_ and FVC in groups of workers employed at various workplaces in the same copper mine. In all studied groups, *s*_*r*_ was lower than referential value, which is 4%. *Z*-score values, presented graphically as charts of trends for FEV_1_ and FVC, were similar in all groups. Nonetheless, although the assessment of respiratory outcomes followed an international standardized protocol, the FEV_1_ (FVC) excessive variance in follow-up study was relatively high.

In our analysis, the lung dysfunction was defined as FEV_1_, FVC, or FEV_1_/FVC ratio lowered below the LLN (5th percentile in the reference population, sex, age, and height matched). Although this method is burdened with a 5% risk of false positive results, such a risk is generally acceptable. This type of presentation is regarded as the gold standard in epidemiological studies and recommended by scientific societies such as ATS, ERS, and BTS [26].

## 5. Conclusions

In long-term follow-up, lung function in copper miners working underground at various workplaces was similar. Nevertheless, in cross-sectional analysis, a significant association between the type of job and lung function appeared after 20 years of work. Additionally, as soon as after 10 years of work, discrete obstructive type disturbances appeared in welders. This may be associated with smoking (the proportion of smokers in this group of miners compared with other groups was higher). Also, welding gases containing nitrogen oxides can affect airway flow in these workers, especially as, after 20 years of work, interactions between workplace and smoking were found. Thus, it should be emphasized that smoking welders should be referred to early clinical diagnostics.

## Figures and Tables

**Table 1:** Characteristics of copper miners monitored by spirometry over 25 years of work. Comparison of the welders group to the group of operators and other miners.

	The first study	After 10 years	After 20 years	After 25 years
All groups (*n*)	888	857	603	739
Age (yr), mean (SD)	29,5 ± 4,2	39,5 ± 4,2	48,7 ± 3,8	54,2 ± 4,1
Height (cm), mean (SD)	174,9 ± 6,0	174,9 ± 6,2	173,9 ± 6,0	175,2 ± 6,3
Weight (kg), median (IQR)	80,0 (72, 88)	82,0 (74, 91)	84,0 (76, 93)	86,0 (79, 95)
BMI (kg/m^2^), median (IQR)	26,1 (24,1, 28,4)	26,7 (24,7, 29,3)	27,7 (25, 30,1)	27,9 (25,8, 30,4)
Smokers (*n*/%)	354/44,9%	371/43,5%	257/42,9%	280/38,0%
Period of smoking (yr)	8,1 ± 5,6	13,1 ± 5,9	18,8 ± 7,0	22,3 ± 7,8
Nonsmokers (*n*/%)	435/55,1%	480/56,5%	341/57,1%	456/62,0%
Respiratory disease: total (coniosis/allergy) (*n*)	15 (8/7)	14 (8/6)	8 (6/2)	15 (8/7)
Operators group (*n*)	225	222	219	144
Age (yr), mean (SD)	26,8 ± 3,2	36,8 ± 3,2	46,9 ± 3,3	52,7 ± 3,7
Height (cm), mean (SD)	174,3 ± 6,1	174,4 ± 6,1	174,7 ± 6,3	174,9 ± 6,0
Weight (kg), median (IQR)	80,0 (71, 88)	82,0 (73, 91)	84,0 (76, 95)	87,0 (80, 96)
BMI (kg/m^2^), median (IQR)	25,8 (23,7, 28,6)	26,7 (24,5, 29,3)	27,5 (25, 29,9)	28,4 (26,1, 30,9)
Smokers (*n*/%)	92/41,3%	97/44,7%	95/45,3%	47/32,6%
Nonsmokers (*n*/%)	132/58,7%	120/55,3%	122/55,7%	97/67,4%
“Others” group (*n*)	475	461	239	428
Age (yr), mean (SD)	30,3 ± 3,9	40,3 ± 3,9	49,9 ± 4,1	53,9 ± 3,8
Height (cm), mean (SD)	174,9 ± 6,2	175,0 ± 6,2	172,6 ± 5,3	175,3 ± 6,2
Weight (kg), median (IQR)	80,0 (72, 88)	82,0 (73, 91)	81,0 (74, 91)	86,0 (78, 95)
BMI (kg/m^2^), median (IQR)	26,1 (24,1, 28,4)	26,5 (24,5, 28,9)	27,5 (25, 30,1)	27,7 (25,8, 30,2)
Smokers (*n*/%)	178/42,3%	191/42,1%	102/42,7%	170/39,5%
Nonsmokers (*n*/%)	243/57,7%	267/57,9%	136/56,9%	257/60,5%
Welders group (*n*)	188	174	145	167
Age (yr), mean (SD)	31,7 ± 4,5^*∗∗*^	41,7 ± 4,5^*∗∗*^	49,3 ± 2,9^*∗∗∗*^	56,4 ± 4,3^*∗∗∗*^
Height (cm), mean (SD)	175,6 ± 5,6	174,8 ± 6,4	174,9 ± 6,5	174,9 ± 6,8
Weight (kg), median (IQR)	83,0 (74, 90)^*∗∗*,°°^	84,0 (77, 91)^*∗∗*,°°^	86,0 (80, 95)^*∗∗*,°°^	85,5 (78, 96)
BMI (kg/m^2^), median (IQR)	27,4 (25,3, 29,1)^*∗∗*,°°^	27,4 (25,3, 27,2)	28,4 (25, 31,1)	28,1 (25,8, 30,3)
Smokers (*n*/%)	84/58,4%°°°	83/46,7%°°°	60/41,9%	63/37,7%°°°
Nonsmokers (*n*/%)	60/41,6%°°°	93/53,3%°°°	83/58,1%°	104/62,3%°°°

^*∗*,*∗∗*,*∗∗∗*^Statistically significant differences between welders and operators groups: ^*∗*^ *p* < 0,05; ^*∗∗*^ *p* < 0,01; ^*∗∗∗*^ *p* < 0,001.

^°,°°°^Statistically significant differences between welders and “other” miners: °*p* < 0,05; °°*p* < 0,01; °°°*p* < 0,001.

**Table 2:** FEV_1_/FVC ratio in the studied groups of copper miners at the start of employment (the first spirometry) and 10, 20, and 25 years later.

	*n*	Mean ± SD	*Q*25	Median	*Q*75	5th percentile	95th percentile
	The first study						
Operators	220	84,7 ± 13,6	78,7	86,0	91,4	63,9	97,4
Others	465	88,8 ± 52,2	81,6	87,2	92,3	69,7	97,8
Welders	178	84,9 ± 8,92	81,3	85,6	90,2	68,0	96,6
Total	863	85,6 ± 11,3	81,2	86,4	91,7	68,0	97,2

	After 10 years						
Operators	221	84,9 ± 8,27	80,5	85,8	89,9	74,9	93,4
Others	456	84,5 ± 52,2	81,6	87,2	92,3	75,6	96,5
Welders	173	84,3 ± 7,34	81,1	85,4	89,1	75,8	92,1
Total	850	84,5 ± 7,60^*∗∗*^	80,5	85,3	89,2	75,5	92,6

	After 20 years						
Operators	219	82,5 ± 7,05	79,2	83,1	86,5	74,3	89,5
Others	239	83,9 ± 8,36	78,9	83,8	87,8	73,9	92,5
Welders	144	83,5 ± 8,22	80,1	83,8	86,6	73,9	91,4
Total	602	83,7 ± 11,8^*∗∗∗*^	79,3	85,6	86,9	74,4	91,1

	After 25 years						
Operators	144	82,5 ± 6,60	79,6	83,1	85,8	73,7	89,6
Others	429	82,8 ± 11,9	78,7	83,4	86,2	73,6	90,7
Welders	166	81,7 ± 6,90	77,8	82,3	85,6	73,3	89,6
Total	739	82,5 ± 10,1^*∗∗∗*^	78,7	85,5	86,0	73,5	90,1

Paired *t*-test for dependent samples. Statistically significant difference in comparison to the results of the first spirometry performed in the group of all miners (total); ^*∗∗*^ *p* < 0,01; ^*∗∗∗*^ *p* < 0,001.

**Table 3:** Association between type of workplace and lung function after 10, 20, and 25 years of professional work. Multiple linear regressions adjusted for age, height, weight, BMI, smoking, and coexistence of respiratory disease.

		After 10 years	After 20 years	After 25 years
Workplace	Level compared	FVC *β*-coefficient (95% CI)	FVC *β*-coefficient (95% CI)	FVC *β*-coefficient (95% CI)
Group	Welders	0	0	0
	Operators	−0,06 (−0,13 to 0,01)	0,17 (−0,18 to 0,52)	−0,09 (−0,21 to 0,03)
	Others	0.06 (−0.01 to 0.13)	−0,56 (−0,90 to −0,22)	0,21 (−0,33 to −0,08)
	*p *value	0.08	**0,0022**	**0,0012**

Workplace	Level compared	FEV_1_ *β*-coefficient (95% CI)	FEV_1_ *β*-coefficient (95% CI)	FEV_1_ *β*-coefficient (95% CI)
Group	Welders	0	0	0
	Operators	−0,04 (−0,12 to 0,02)	0,23 (−0,10 to 0,57)	−0,04 (−0,24 to 0,008)
	Others	0,05 (−0,012 to 0,13)	−0,63 (−0,95 to −0,30)	−0,12 (−0,95 to −0,30)
	*p *value	0,17	**0,0004**	0,0667

Workplace	Level compared	FEV_1_/FVC *β*-coefficient (95% CI)	FEV_1_/FVC *β*-coefficient (95% CI)	FEV_1_/FVC *β*-coefficient (95% CI)
Group	Welders	0	0	0
	Operators	0,02 (−0,06 to 0,09)	0,19 (−0,24 to 0,64)	0,04 (0,09 to −0,17)
	Others	−0,005 (−0,08 to 0,06)	−0,01 (−0,43 to 0,41)	0,09 (−0,03 to 0,22)
	*p *value	0,61	0,37	0,14

Workplace	Level compared	PEF *β*-coefficient (95% CI)	PEF *β*-coefficient (95% CI)	PEF *β*-coefficient (95% CI)
Group	Welders	0	0	0
	Operators	−0,001 (−0,08 to 0,07)	0,02 (−0,36 to 0,40)	0,004 (−0,12 to 0,13)
	Others	0,04 (−0,03 to 0,11)	−0,31 (−0,68 to 0,40)	−0,13 (−0,25 to 0,007)
	*p *value	0,28	0,08	**0,0379**

Workplace	Level compared	FEV_25_ *β*-coefficient (95% CI)	FEV_25_ *β*-coefficient (95% CI)	FEV_25_ *β*-coefficient (95% CI)
Group	Welders	0	0	0
	Operators	0,04 (−0,03 to 0,11)	0,02 (−0,40 to 0,45)	−0,02 (−0,15 to 0,10)
	Others	−0,06 (−0,13 to 0,01	−0,24 (−0,66 to 0,16)	−0,07 (−0,20 to 0,05)
	*p *value	0,09	0,22	0,23

Workplace	Level compared	FEV_25–75_ *β*-coefficient (95% CI)	FEV_25–75_ *β*-coefficient (95% CI)	FEV_25–75_ *β*-coefficient (95% CI)
Group	Welders	0	0	0
	Operators	−0,01 (−0,09 to 0,06)	0,34 (−0,06 to 0,75)	0,06 (−0,06 to 0,19)
	Others	0,02 (−0,05 to 0,09)	−0,44 (−0,83 to −0,05)	−0,01 (−0,14 to 0,11)
	*p *value	0,58	**0,0268**	0,29

Workplace	Level compared	FEV_50_ *β*-coefficient (95% CI)	FEV_50_ *β*-coefficient (95% CI)	FEV_50_ *β*-coefficient (95% CI)
Group	Welders	0	0	0
	Operators	−0,02 (−0,09 to 0,05)	0,39 (0,009 to 0,78)	0,01 (−0,11 to 0,14)
	Others	0,03 (−0,04 to 0,10)	−0,41 (−0,79 to −0,04)	0,01 (−0,12 to0,14)
	*p *value	0,49	**0,0282**	0,83

Workplace	Level compared	FEV_75_ *β*-coefficient (95% CI)	FEV_75_ *β*-coefficient (95% CI)	FEV_75_ *β*-coefficient (95% CI)
Group	Welders	0	0	0
	Operators	−0,03 (−0,11 to 0,04)	0,04 (−0,37 to 0,46)	0,07 (−0,05 to 0,20)
	Others	0,07 (0,001 to 0,15)	0,03 (−0,36 to 0,44)	0,002 (−0,12 to 0,13)
	*p *value	0,44	0,83	0,23

CI: confidence interval; *p* values in bold indicate statistical significance.

**Table 4:** The effect of workplace and smoking and interaction between these factors in their effect on spirometric parameters in copper miners after 10, 20, and 25 years of work.

Effect	After 10 years		After 20 years		After 25 years	
	*F*	*p*	*F*	*p*	*F*	*p*
	% FVC		FVC		FVC	
Workplace	0,72	0,4859	5,85	**0,0075**	8,32	**0,0003**
Smoking	1,77	0,1840	2,04	0,1633	0,04	0,8376
Workplace *∗* smoking	0,30	0,7391	4,22	**0,0248**	1,90	**0,0089**

	% FEV_1_		FEV_1_		% FEV_1_	
Workplace	0,91	0,4017	8,02	**0,0017**	0,83	0,4331
Smoking	0,15	0,6957	5,03	**0,0328**	1,97	0,1611
Workplace *∗* smoking	0,10	0,9088	5,02	**0,0136**	0,52	0,5934

	FEV_1_/FVC		FEV_1_/FVC		FEV_1_/FVC	
Workplace	0,39	0,6753	0,17	0,8385	2,36	0,0964
Smoking	0,79	0,3730	1,40	0,2463	4,32	**0,0387**
Workplace *∗* smoking	1,10	0,3339	0,29	0,7499	1,24	0,2901

	% PEF		% PEF		% PEF	
Workplace	1,62	0,1972	1,11	0,3431	1,94	0,1457
Smoking	0,02	0,8768	0,26	0,6100	6,67	**0,0102**
Workplace *∗* smoking	0,50	0,6033	3,70	**0,0373**	1,76	0,1736

	% FEF_25_		% FEF_25_		% FEF_25_	
Workplace	0,65	0,5220	1,41	0,2593	0,36	0,6958
Smoking	1,72	0,1900	4,09	0,0525	3,33	0,0691
Workplace *∗* smoking	0,28	0,7534	3,44	**0,0460**	0,75	0,4725

	% FEF_50_		FEF_50_		% FEF_50_	
Workplace	0,44	0,6382	3,38	**0,0481**	0,33	0,7188
Smoking	0,13	0,7133	2,73	0,1096	2,81	0,0944
Workplace *∗* smoking	0,76	0,4638	3,84	**0,0334**	0,64	0,5275

	% FEF_75_		% FEF_75_		% FEF_75_	
Workplace	0,62	0,5362	0,39	0,6775	0,95	0,3878
Smoking	0,29	0,5899	1,67	0,2067	4,48	**0,0350**
Workplace *∗* smoking	1,74	0,1750	3,35	**0,0494**	0,54	0,5819

Spreadsheet of two-way analysis of variance; one-dimensional tests of significance. *F*: *F*-test value for the respective effects; *p*: the probability level of *p*; values indicated in bold are statistically significant.

## Abbreviations

FVC: Forced vital capacity
FEV_1_: Forced expiratory volume in the first second of forced exhalation
FEF_25%_: Forced expiratory flow at 25% of expired forced vital capacity
FEF_50%_: Forced expiratory flow at 50% of expired forced vital capacity
FEF_75%_: Forced expiratory flow at 75% of expired forced vital capacity
FEF_25%–75%_: Forced expiratory volume between 25% and 75% of FVC
PEF: Peak expiratory flow.
